# Longitudinal Analysis of Peripheral Blood CD4+ T-Cell Profiles and Clinical Outcomes in Metastatic Non-Small-Cell Lung Cancer Patients Following Bronchoscopic Cryotherapy and Pembrolizumab-Based Therapy

**DOI:** 10.3390/ijms27072927

**Published:** 2026-03-24

**Authors:** Gediminas Vasiliauskas, Evelina Žemaitė, Erika Skrodenienė, Lina Poškienė, Skaidrius Miliauskas, Marius Žemaitis

**Affiliations:** 1 Department of Pulmonology, Lithuanian University of Health Sciences, 44307 Kaunas, Lithuania; skaidrius.miliauskas@lsmu.lt; 2 Department of Laboratory Medicine, Lithuanian University of Health Sciences, 44307 Kaunas, Lithuania; evelina.zemaite@lsmu.lt (E.Ž.); erika.skrodeniene@lsmu.lt (E.S.); 3 Department of Pathology, Lithuanian University of Health Sciences, 44307 Kaunas, Lithuania; lina.poskiene@lsmu.lt

**Keywords:** non-small-cell lung cancer, cryotherapy, immunotherapy, flow cytometry, CD4+ T cells

## Abstract

Bronchoscopic cryotherapy is routinely used for endobronchial tumor debulking, but may also exert systemic immunologic effects that could interact with immune checkpoint blockade. We investigated peripheral blood T-cell dynamics following bronchoscopic cryotherapy and subsequent pembrolizumab-based first-line therapy in metastatic non-small-cell lung cancer (NSCLC). In this prospective, randomized, controlled single-center study, patients with metastatic NSCLC were randomized into treatment groups of bronchoscopic cryotherapy performed 7 (±1) days before standard-of-care pembrolizumab (with or without platinum-based chemotherapy) or to standard-of-care therapy alone. Peripheral blood mononuclear cells were analyzed by flow cytometry at baseline, week 3, and week 6. Radiologic response was assessed using RECIST 1.1. Progression-free survival (PFS) and overall survival (OS) were evaluated using the Kaplan–Meier test and Cox regression. Flow cytometry was performed on 34 cryotherapy and 42 control patients. The cryotherapy group demonstrated a decrease in circulating CD4+ T cells (*p* = 0.002) and an increase in circulating CD8+ T cells (*p* = 0.013) by week 6. CD25+FOXP3+CD4+ Tregs decreased from baseline to week 3 (*p* = 0.024) and remained reduced through week 6. Overall response rate was higher in the cryotherapy group (41.2% vs. 16.7%; *p* = 0.022), while PFS and OS were numerically longer, although not statistically different (median PFS 9.5 vs. 5.3 months; median OS 17.6 vs. 14.8 months). The decrease in Tregs at week 3 was observed to predict better PFS. In patients with metastatic NSCLC receiving first-line pembrolizumab with or without chemotherapy, the addition of bronchoscopic cryotherapy was associated with a detectable peripheral immune remodeling and a higher objective response rate, whereas PFS and OS were numerically longer but not statistically different.

## 1. Introduction

Lung cancer remains a significant global health challenge, which accounted for roughly 2.5 million new cases and approximately 1.8 million deaths worldwide in 2022 [1]. Lung cancer comprises several histologic entities, of which non-small-cell lung cancer (NSCLC) represents the majority of cases.

Over the past decade, immune checkpoint inhibitors (ICIs) have become an important therapeutic modality for advanced NSCLC. For example, randomized trials have shown that ICI pembrolizumab, which targets the programmed cell death protein 1/programmed death-ligand 1 (PD-1/PD-L1) axis, can provide durable progression-free and overall survival compared with platinum-based chemotherapy in patients with high PD-L1 expression [2]. Nevertheless, a substantial number of patients receiving ICIs experience either no benefit or eventual progression, providing further impetus to both search for ways to improve efficacy and to better understand prognostic markers of response or resistance [3,4].

While much of the biomarker research has centered on tumor programmed death-ligand 1 (PD-L1) expression and CD8+ effector cells, CD4+ T cells are increasingly recognized as critical mediators of anti-tumor immunity and immunotherapy outcomes. CD4+ T cells may support anti-tumor activity but also restrain it and impair ICI efficacy by promoting immunosuppressive regulatory T cells (Tregs) [5,6]. The longitudinal shifts in circulating T cells have been investigated as candidate biomarkers of response or disease progression during PD-1/PD-L1 blockade [7,8]. However, the dynamic changes in CD4+ T cells and their subpopulations following bronchoscopic cryotherapy combined with immunotherapy have not been well characterized. Bronchoscopic cryotherapy is widely used in interventional pulmonology for endobronchial tumor debulking and airway recanalization. Furthermore, it may also provide immunologic effects beyond local cytoreduction [8,9]. Mechanistically, cryotherapy induces tumor cell death and promotes antigen release and inflammatory signaling, with preclinical and translational studies supporting its capacity to activate dendritic cell–dependent pathways and potentiate responses to PD-1 blockade [10,11]. The current cryotherapy research landscape is limited to several small studies, necessitating larger-scale controlled studies [12,13].

Accordingly, we evaluated early dynamics of peripheral blood T-cell populations in patients with metastatic NSCLC treated with bronchoscopic cryotherapy followed by pembrolizumab immunotherapy alone or in combination with chemotherapy. In this paper, we focused on CD4+ T-cell subpopulations, with CD8+ T lymphocytes being described more thoroughly in an upcoming publication. We aimed to describe post-cryotherapy systemic CD4+ remodeling and lay the groundwork for exploring whether specific CD4+ subpopulation changes are associated with different clinical outcomes.

## 2. Results

### 2.1. Patient Characteristics and Their Association with Peripheral Blood T Cells

**Figure 1:**
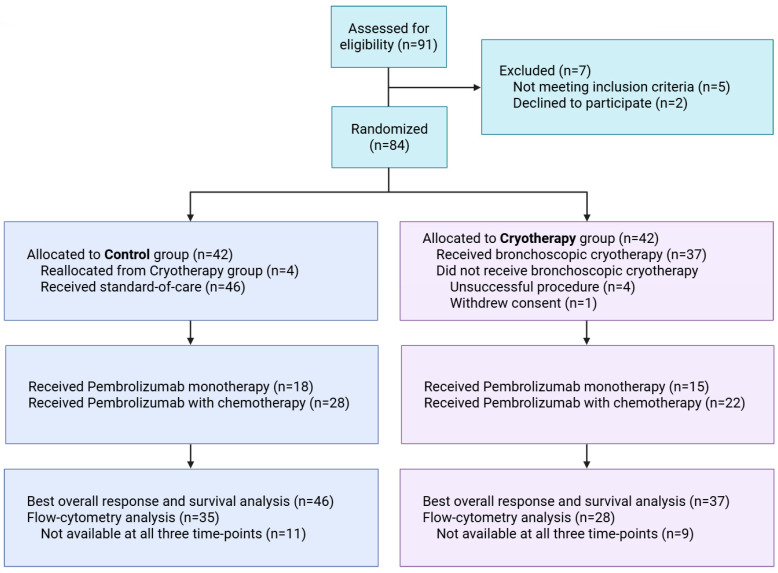
CONSORT diagram.

In total, 83 patients were included in our study; however, due to delayed material procurement, flow cytometry could only be performed for 76 patients, with 34 in the cryotherapy group and 42 in the control group (Figure 1). Four patients who were initially assigned to the cryotherapy group had an unsuccessful procedure and were reassigned to the control group, with reasons for failure stated in our earlier paper [7]. Baseline demographic and disease characteristics were broadly comparable between groups (Table 1). The median age of all included patients was 65 (60–72) years; most (77.6%) patients were male. Squamous histology was present in 44.7% of cases, with the remaining 55.3% being adenocarcinomas. PD-L1 expression of <1%, 1–49%, and ≥50% was observed in 34.2%, 25.0%, and 40.8% of patients, respectively, translating to 40.8% of patients receiving pembrolizumab monotherapy and 59.2% of patients receiving pembrolizumab with platinum-based chemotherapy.

At week 3, paired samples were available for most patients (30 for cryotherapy and 35 for the control group). By week 6, additional attrition occurred (28 patients remaining in cryotherapy and 35 in the control group). Patients were unable to provide subsequent samples due to deteriorating functional status or death, with four patients dying in each group. No significant differences in T-cell populations were observed depending on clinical characteristics, such as age, gender, smoking history, Eastern Cooperative Oncology Group (ECOG) performance status, histology, or systemic treatment modality at the beginning of the study (Supplemental Table S1).

### 2.2. Changes in Peripheral Blood T Cells

**Figure 2:**
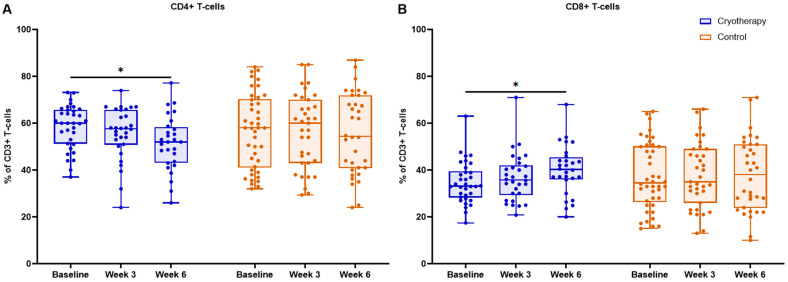
Dynamics of peripheral blood (**A**) CD4+ and (**B**) CD8+ T cells, reported as a percentage of CD3+ T cells, in cryotherapy and control groups at baseline, week 3, and week 6. Boxes indicate median and interquartile range; points represent individual patients; * *p* < 0.05.

The whole population of CD3+ T cells, as a proportion of all lymphocytes, remained stable in both the cryotherapy (74.0 vs. 72.0 vs. 71.0%) and control (69.0 vs. 72.0 vs. 73.0%) groups, and did not change either longitudinally or between groups (all *p* > 0.05). We further assessed the dynamic changes in the relative abundance of circulating CD4+ and CD8+ T cells. There was a longitudinal decline in CD4+ T cells in the cryotherapy group, achieving statistical significance by week 6 compared to baseline (*p* = 0.002) (Table 2, Figure 2). In turn, CD8+ T cells in the cryotherapy group demonstrated an upward trend, with a statistically significant increase from baseline to week 6 (*p* = 0.013). In the control group, CD4+ and CD8+ T cells did not change significantly at any point (within-group *p* > 0.05).

There were no significant differences in CD4+ and CD8+ T-cell levels between the cryotherapy and control groups at baseline, week 3, or week 6 (Table 2, Figure 1). As this paper focuses on CD4+ T cells, with CD8+ T cells examined more thoroughly in an upcoming publication, additional CD4+ T-lymphocyte subpopulations were analyzed and described as percentages of CD4+ T cells.

### 2.3. Changes in Peripheral CD4+ T-Cell Subpopulations

No significant longitudinal changes were observed in the frequencies of CD4+T-bet+ (Th1), CD4+GATA3+ (Th2), or CD4+RORγt+ (Th17) cells within either study group (all within-group *p* > 0.05) (Table 3). Furthermore, no inter-group differences were detected at baseline, week 3, or week 6 for these cells, except that Th2 and Tregs were higher at baseline in the cryotherapy group compared with controls (*p* = 0.004 and *p* = 0.017, respectively).

**Figure 3:**
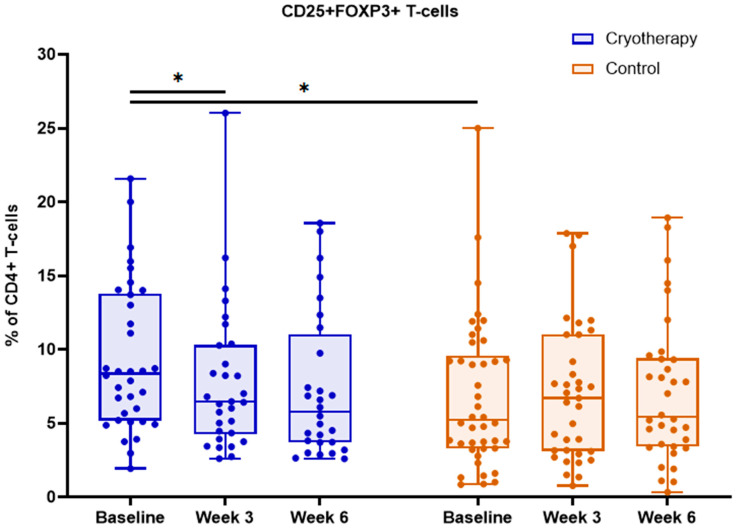
Dynamics of peripheral blood CD4+CD25+FOXP3+ Treg population reported as a percentage of CD4+ T cells in cryotherapy and control groups at baseline, week 3, and week 6. Boxes indicate median and interquartile range; points represent individual patients; * *p* < 0.05.

In the cryotherapy group, Tregs decreased significantly from baseline to week 3 (*p* = 0.024), and remained reduced through to week 6. No significant temporal changes were observed in the control group (Table 3, Figure 3).

### 2.4. Correlation of Cryotherapy Addition and Peripheral Blood T-Cell Profiles with Clinical Outcomes in Patients with NSCLC

At our current cutoff of 1 January 2026, the median follow-up time was 19.8 months (95% CI, 14.3–27.1) based on a reverse Kaplan–Meier approach and overall survival (OS) follow-up. At this time point, 9 (26.4%) patients in the cryotherapy group and 6 (14.3%) patients in the control group were still continuing first-line therapy. Meanwhile, 14 patients remained alive in each group (41.2% and 33.3%, respectively).

**Figure 4:**
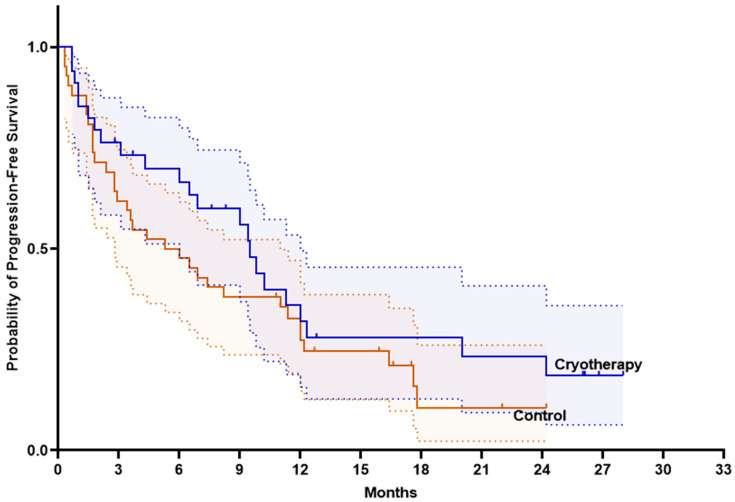
Progression-free survival in the intention-to-treat population. Straight line represents survival probability; dashed lines represent 95% confidence intervals.

**Figure 5:**
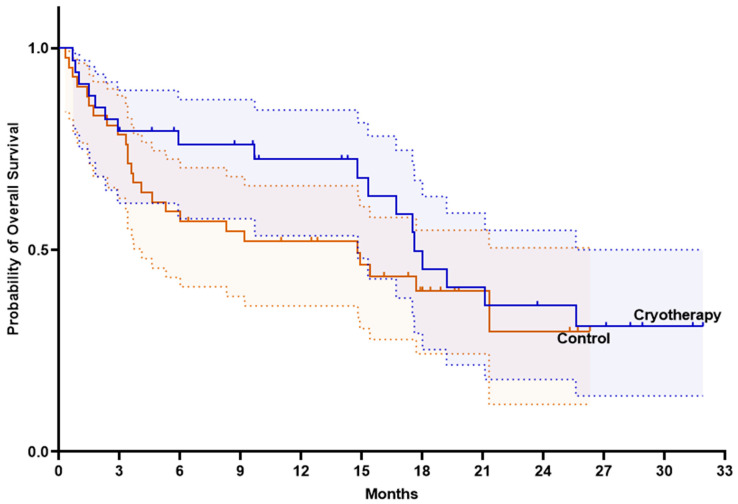
Overall survival in the intention-to-treat population. Straight line represents survival probability; dashed lines represent 95% confidence intervals.

Initially, Kaplan–Meier survival curves were used to assess whether the addition of cryotherapy to immunotherapy influenced progression-free survival (PFS) and OS. PFS and OS were numerically higher in the cryotherapy group compared with the control group, although the difference was not statistically significant. Median PFS was 9.5 months (95% CI 8.3–10.8) in the cryotherapy group and 5.3 months (95% CI 1.7–8.8) in the control group (log-rank *p* = 0.237) (Figure 4). Meanwhile, median OS was 17.6 months (95% CI 14.9–20.3) in the cryotherapy group versus 14.8 months (95% CI 4.1–25.4) in the control group (log-rank *p* = 0.345) (Figure 5). No significant differences in survival were observed between the cryotherapy and control groups among patients receiving either pembrolizumab monotherapy or pembrolizumab plus chemotherapy (*p* > 0.05).

**Figure 6:**
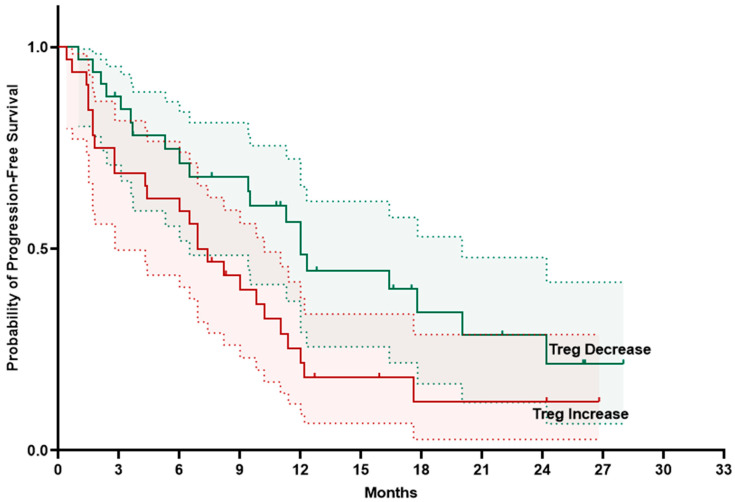
Progression-free survival according to Treg changes at week 3. Straight line represents survival probability; dashed lines represent 95% confidence intervals.

Analysis of the Kaplan–Meier survival curves for peripheral blood T cells, CD4+ subpopulations, and their longitudinal changes revealed that only the change in Treg population was significantly associated with PFS. The median PFS was 12.0 months (95% CI 10.6–13.4) in patients with NSCLC who exhibited a decrease in Tregs at week 3, compared with 6.9 months (95% CI 4.6–9.2) in patients who had a Treg increase (log-rank *p* = 0.032) (Figure 6).

Subsequent univariate and multivariable Cox regression analyses were performed to identify potential predictors of PFS and OS, with results presented in Table 4 and Table S2. Multivariate analysis identified a reduction in Treg cells at week 3 as an independent predictor of improved PFS, as well as PD-L1 > 50% expression as an independent predictor of improved PFS and OS.

Best overall response according to Response Evaluation Criteria In Solid Tumors version 1.1 (RECIST 1.1) criteria during follow-up is provided in Table 5. The overall distribution of responses did not differ significantly between groups (χ^2^ = 5.71; df = 2; *p* = 0.063). Disease control rate (DCR) was also similar between groups (73.5% vs. 59.5%; χ^2^ = 1.64; df = 1; *p* = 0.232). However, overall response rate (ORR) was higher in the cryotherapy group (41.2% vs. 16.7%; χ^2^ = 5.65; df = 1; *p* = 0.022). This result was mainly driven by patients with a low PD-L1 tumor proportion score (TPS), as the differences in ORR between cryotherapy and control groups were not significant in patients receiving pembrolizumab monotherapy (40.0% vs. 25.0%; χ^2^ = 0.80; df = 1; *p* = 0.458), contrary to those receiving pembrolizumab with chemotherapy (42.1% vs. 11.5%; χ^2^ = 5.55; df = 1; *p* = 0.033).

**Figure 7:**
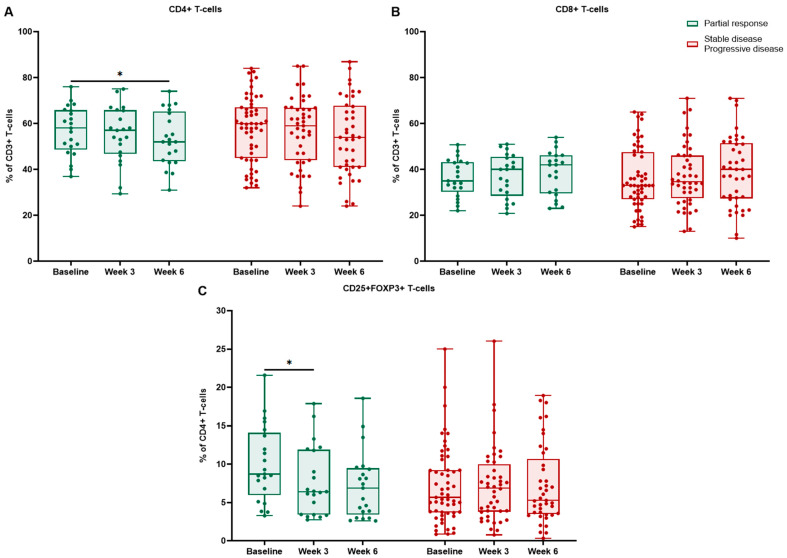
Peripheral blood T-cell dynamics by radiologic response among responders vs. non-responders: CD4+ T cells (**A**) and CD8+ T cells (**B**) reported as a percentage of CD3+ T cells, CD4+CD25+FOXP3+ Tregs (**C**) reported as a percentage of CD4+ T cells at baseline, week 3, and week 6. Boxes indicate median and interquartile range; points represent individual patients; * *p* < 0.05.

**Figure 8:**
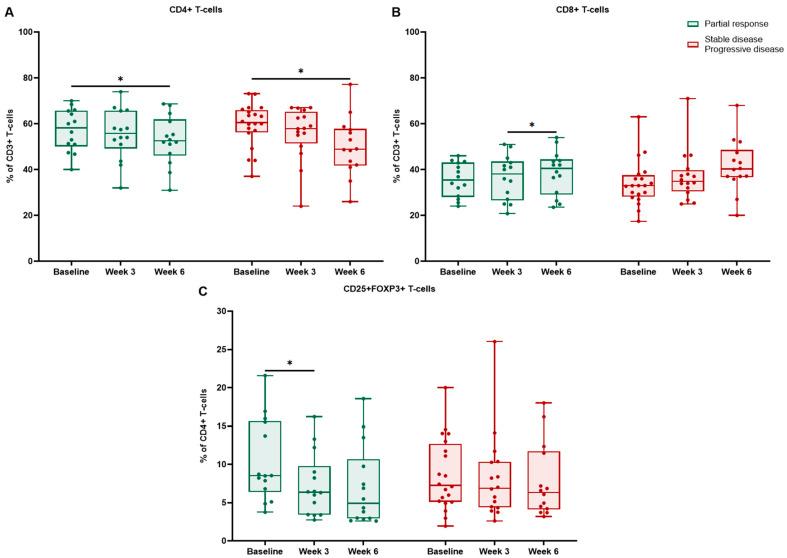
Peripheral blood T-cell dynamics by radiologic response among responders vs. non-responders in the cryotherapy group: CD4+ T cells (**A**) and CD8+ T cells (**B**) reported as a percentage of CD3+ T cells, CD4+CD25+FOXP3+ Tregs (**C**) reported as a percentage of CD4+ T cells at baseline, week 3, and week 6. Boxes indicate median and interquartile range; points represent individual patients; * *p* < 0.05.

We further analyzed flow cytometry results in different response groups. Only the partial response (PR) subgroup demonstrated significant longitudinal changes (Table 6). In patients achieving PR, CD4+ T cells showed a decreasing trend, reaching statistical significance between baseline and week 6 (*p* = 0.023) (Figure 7). This was driven mainly by the cryotherapy group, where among responders and non-responders, CD4+ T cells declined significantly from baseline to week 6 (*p* = 0.019 and *p* = 0.049, respectively) (Figure 8). In patients with stable disease (SD) or progressive disease (PD), the CD4+ population showed no significant decrease (all *p* > 0.05).

For CD8+ T cells, patients with PR showed a numerical increase over time; however, no significant change was observed in either group (all *p* > 0.05). Nevertheless, among cryotherapy responders, CD8+ T cells reached significance at week 6 (*p* = 0.033). In comparison, cryotherapy non-responders (SD/PD) showed a numerical increase in CD8+ T cells, with a trend from baseline to week 6 (*p* = 0.064).

Tregs diminished from baseline to week 3 in responders (*p* = 0.029) (Figure 7). Among cryotherapy responders, Tregs decreased significantly from baseline to week 3 (*p* = 0.007) and remained numerically lower at week 6. In comparison, cryotherapy non-responders showed no significant changes in Tregs (all *p* > 0.05).

**Figure 9:**
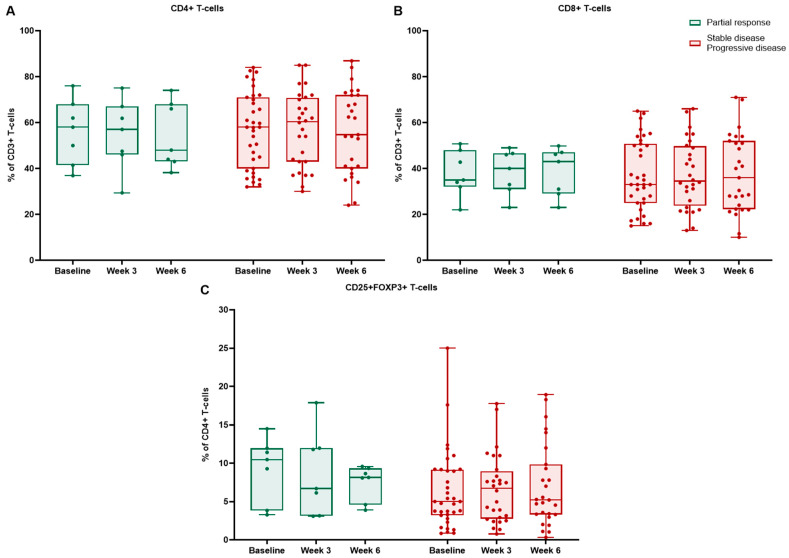
Peripheral blood T-cell dynamics by radiologic response among responders vs. non-responders in the control group. CD4+ T cells (**A**) and CD8+ T cells (**B**) reported as a percentage of CD3+ T cells, CD4+CD25+FOXP3+ Tregs (**C**) reported as a percentage of CD4+ T cells at baseline, week 3, and week 6. Boxes indicate median and interquartile range; points represent individual patients.

Neither responders nor non-responders in the control group demonstrated significant longitudinal changes in CD4+, CD8+, or Treg proportions (all within-group *p* > 0.05); however, responders in the control group constituted only seven patients (Figure 9). Of these, six and three patients had a decrease in CD4+ T cells from baseline to 3 and 6 weeks, respectively, while four and three had an increase in CD8+ at these time points. In total, five patients had a decrease in FOXP3 at week 3, maintaining it to week 6. No significant changes were observed for Th1, Th2, and Th17 T cells in either response group (Supplemental Table S3).

## 3. Discussion

In this randomized controlled study of patients with metastatic NSCLC receiving first-line pembrolizumab (with or without chemotherapy), bronchoscopic cryotherapy was associated with a measurable remodeling of peripheral T-cell compartments during the first 6 weeks of systemic therapy, leading to improved ORR; however, PFS and OS differences were not statistically significant. To our knowledge, this is the first prospective study examining peripheral T-cell changes in patients with NSCLC receiving a combination of cryotherapy and immunotherapy. However, several murine studies have shown that cryotherapy-induced tumor cell death can release neoantigens and danger signals that promote a tumor-specific T-cell response [9,10,11]. Liu et al. evaluated the effects of combined cryotherapy and immunotherapy in a murine Lewis lung adenocarcinoma model, finding an increase in both tumor-infiltrating and circulating CD8+ T cells, as well as a reduction in Tregs, similar to our study [10]. These changes were linked to the PI3K/AKT/mTOR pathway, which is triggered by antigen recognition and inhibits Treg induction while promoting effector CD8+ T cells [14]. While both cryotherapy and immunotherapy alone appeared to activate this pathway compared with controls, the greatest benefit was observed with both treatments combined. Cryotherapy may also be superior in inducing this pathway compared to heat-based therapies due to a reversible denaturation of tumor cell proteins, which retain their structure and complete immunogenicity [15]. Another pathway, which was observed to be activated in cryoablated murine tumors, is the stimulator of interferon (STING) [11]. This pathway is induced by the cytosolic DNA from destroyed tumor cells and is critical for type I interferon production. In mouse models, type I interferons—such as interferon-α (IFN-α)—suppress the production of CCL17, an attractant of Tregs, therefore suppressing their trafficking into the tumor microenvironment [16]. Furthermore, type I interferons have been shown to induce PD-1/PD-L1 expression, which in turn increases the susceptibility of cancer cells to immunotherapy, providing a basis for the synergistic effect of these therapies [17,18]. Finally, single-cell RNA sequencing shows that cryoablation inhibits the TGF-β/SMAD/FOXP3 pathway [19]. Transforming growth factor-β (TGF-β)—produced in the tumor microenvironment by platelets, fibroblasts, tumor-associated macrophages, and even cancer cells themselves—forms the SMAD complex and activates forkhead box protein P3 (FOXP3), generating Tregs from naive T cells [19,20].

The T-cell changes observed in preclinical studies have been confirmed by several small studies of patients with NSCLC [11,12]. Tsay et al. performed bronchoscopic cryotherapy on peripheral lung tumors in 21 patients with advanced NSCLC [12]. The authors observed a decrease in proliferating (Ki-67+) Tregs as a percentage of all Tregs on day 7, with a subsequent increase from this low point to day 14. Similarly, Gu et al. evaluated the peripheral blood samples of six patients with NSCLC treated with percutaneous cryoablation, finding a reduction in the proportion of Tregs in four of them [11]. Cryoablation studies of other cancers have reported similar results. For example, Pediconi et al. observed a significant decrease in Ki-67+ Tregs within 21 days after cryoablation in early-stage breast cancer. Meanwhile, we found that combined cryotherapy and immunotherapy treatment led to a decrease in Tregs at week 3 compared to baseline. While not directly comparable, the results of previous studies, combined with our findings, suggest that cryotherapy itself negatively affects the Treg population, with subsequent immunotherapy maintaining the new equilibrium. Another important factor to consider is that the intensity of cryotherapy and the subsequent load of released neoantigens may elicit a different effect on T-cell proliferation. Further research with larger sample sizes is necessary to determine what optimal length of freezing induces sustainable T-cell changes with the best clinical outcomes. The effects of cryotherapy may indeed be “dose-dependent”, as some authors suggest maintaining a –40 °C temperature—lethal for cells—for at least 1 min per cycle, with others providing evidence for durations twice that much [21,22,23].

We observed no changes in T-cell populations in the control group. Although these findings are consistent with several other studies, the current research landscape is quite heterogeneous. Several studies found no differences in CD4+ and CD8+ populations during treatment with a PD-1 inhibitor alone or in combination with platinum-based chemotherapy [24,25,26]. In contrast, Gelibter et al. observed T-cell dynamics in 48 patients with metastatic NSCLC receiving ICIs as monotherapy or in combination with chemotherapy [27]. With measurements made at baseline and week 3 of systemic therapy, a significant reduction in the CD4+ subset (as a percentage of CD3+ T cells) was observed. Gelibter et al. postulated that immunotherapy largely acts on cytotoxic or CD8+ T cells, enhancing their proliferation and migration to the tumor site, therefore decreasing the relative CD4+ proportion [27]. While the inhibition of PD-1/PD-L1 axis is indeed primarily aimed at activating cytotoxic CD8+ T cells, it is likely insufficient at inducing consistent and reproducible changes in peripheral blood, as yet another study observed opposite dynamics, with increased CD4+ T-cell counts and a higher CD4+/CD8+ ratio after PD-1 inhibitor treatment [28]. The inconsistent findings may also be related to differences in systemic treatments, as well as evaluation time points and methods. Similarly, inconsistent data have been reported on CD4+ T-cell subpopulations, including Tregs. In vitro studies have shown that pembrolizumab has minimal effect on Treg proliferation, despite a high PD-1 expression of CD4+CD25+ T cells [29,30]. However, PD-1 blockade may lead to secondary changes in this subpopulation due to immune system activation, with most research focusing on Treg changes according to response.

In our current analysis, the RECIST 1.1 assessment showed a better response in the cryotherapy group, with an ORR of 41.2% versus 16.7% in the control group. The PEMBRO-RT phase 2 randomized clinical trial [NCT02492568], where pembrolizumab was combined with a different local tumor destruction modality—stereotactic body radiotherapy—observed a similarly noticeable change in ORR at 12 weeks. Overall, 36% of patients who underwent radiotherapy had a response, compared to 18% in the control group [31]. The researchers noted the largest benefit from radiotherapy in patients with PD-L1 negative tumors, while we observed improved ORR for cryotherapy patients with PD-L1 TPS of less than 50%. More recent translational work from the same study demonstrated systemic immune activation signatures after pembrolizumab plus radiotherapy, including changes in immune gene expression and peripheral immune cell composition, consistent with conversion of immunologically “cold” tumors toward a more inflamed state susceptible to ICIs [32]. Together, these comparisons support the idea that local tumor destruction may increase tumor antigen availability and inflammatory signaling, amplifying systemic therapy effects at the beginning of treatment in an otherwise unfavorable tumor microenvironment. Further patient survival may be related more to heterogeneity in disease burden, treatment beyond progression, or limited study power, as we observed no significant differences in PFS and OS; however, a trend towards better outcomes was present in the cryotherapy group. Meanwhile, the CRYOVATE pilot study [NCT04793815], with cryoactivation performed 5 days prior to pembrolizumab immunotherapy, reported an ORR of 25% with median PFS at 3.8 months and median OS at 13.0 months [13]. These findings differ from our study, where median PFS and OS were 9.5 and 17.6 months, respectively. However, the CRYOVATE study focused on patients with PD-L1 TPS ≥ 50%, while both the PEMBRO-RT study and ours found that the greatest clinical benefit was observed in patients with lower PD-L1 scores. This supports the idea that local tumor destruction is not necessary for all patients, but may be beneficial in select cases, where the response induced by immunotherapy is insufficient.

Several factors were identified as predictors of clinical outcomes in our study. PD-L1 TPS was an independent predictor of both PFS and OS. This is also evident from the available clinical data, as first-line ICI monotherapy was found to be associated with 1-year PFS rates of 40.3%, 35.0%, and 19.9% in PD-L1 TPS groups of ≥50%, 1–49%, and <1%, respectively, with 2-year OS rates being 47.5%, 34.9%, and 16.7% [33]. Regardless of additional chemotherapy, the PD-L1 TPS < 1% is worse in terms of PFS and OS rates (respectively, at 6.2–6.3 and 15.0–17.2 months) compared to PD-L1 TPS 1–49% (respectively, 8.2–8.4 and 18.0–21.8 months) for both squamous and non-squamous cancers [34,35].

The importance of T-cell dynamics during treatment as a predictor of clinical response has been underscored by numerous studies. The earlier-mentioned immunotherapy study by Gelibter et al. observed a decrease in CD4+ and an increase in CD8+ cells to be most prevalent in responders [27]. We observed similar results when observing the whole patient cohort, although these changes became significant later, at week 6, compared to week 3. Interestingly, when comparing responders vs. non-responders in the cryotherapy group alone, both subgroups showed similar changes. Given that most responders were in the cryotherapy group, these changes are likely due to cryotherapy activating the immune pathways discussed earlier, with little effect on overall clinical outcomes.

In our observation, the Treg changes, rather than broad T-cell populations, appear to be more closely related to clinical outcomes. The cryotherapy group in our study presented with a higher proportion of Tregs at the beginning of treatment. However, this could be interpreted as a negative prognostic indicator in this group. For example, evaluating peripheral blood samples of 126 patients with NSCLC, Kagamu et al. reported that the percentage of Treg cells in the total population of CD4+ cells was significantly higher in non-responders to nivolumab immunotherapy [36]. Meanwhile, opposite findings were observed in another study by Gelibter et al., where higher baseline Tregs were associated with better survival [37]. There are varying data regarding Treg changes and clinical outcomes in patients with NSCLC receiving immunotherapy. Kang et al. analyzed blood samples from 74 patients with advanced NSCLC at baseline and on day 7 after initiation of anti-PD-1/PD-L1 therapy. The team found that circulating CD4+CD25+CD127^low^FOXP3+ Tregs, as a percentage of CD4+ T cells, decreased in responders and pseudoprogression but increased in cases of hyperprogression [8]. However, an analysis of 132 patients with advanced NSCLC treated with anti-PD-1 immunotherapy, performed by Koh et al., reported that higher circulating CD4+CD25+CD45RA-FOXP3+ effector Treg frequencies one week after anti-PD-1 immunotherapy correlated with improved outcomes, and was interpreted as a possible homeostatic counterweight to therapy-induced immune activation [38]. Meanwhile, another study showed no changes in the proportion of total circulating Tregs at week 3 of immunotherapy, which is consistent with our observations in the control group [27]. The same study further observed a significant increase in the non-suppressive (CD4+CD25^low^FOXP3^low^CD45RA-) Treg subset and a decrease in the active (CD4+CD25^high^FOXP3^high^CD45RA-) Treg population, resulting in more favorable outcomes. The authors suggest that the immune system activation by ICIs could also influence the development of Tregs toward a less suppressive phenotype. In general, Tregs appear to inhibit antitumor immunity in various solid tumors, including lung cancer, and depletion of Tregs seems to improve the immune response [39].

To our knowledge, no other studies have evaluated the Treg dynamics during treatment as predictors of clinical outcomes in cancer patients receiving cryotherapy combined with immunotherapy. Nevertheless, our findings, along with preclinical studies, support the hypothesis that an early decline in circulating Tregs may contribute to improved anti-tumor responses. As discussed above, cryotherapy can promote antigen release and immune priming, potentially enhancing antigen-specific cytotoxic T-cell activation while simultaneously reducing the relative abundance of immunosuppressive Tregs. Acting together, these effects may shift the systemic balance toward effective anti-tumor immunity. Importantly, although reduced Treg numbers are generally associated with a higher risk of autoimmunity, cryotherapy may help focus immune activation toward tumor antigens, enhancing anti-tumor responses without necessarily increasing off-target immune-related adverse events. However, further research is needed to explore this hypothesis.

Our study has several limitations. Firstly, the relatively small sample size, together with additional attrition during blood sample collection, may have reduced our ability to detect significant changes in T-cell populations. This was especially evident among patients with progressive disease, as a substantial proportion of them died during the first 6 weeks. However, their rapid clinical deterioration may also indicate a tumor burden already too advanced to be effectively treated. Secondly, T-cell quantification without deeper subset resolution, such as activated, resting/naïve, or exhausted T cells, limits cross-study comparability and may partly explain why some ICI studies associate lower Treg frequencies with benefit while others point to the opposite. Nonetheless, we believe that our findings provide a basis for further studies with larger patient pools and a more comprehensive analysis of the immune landscape. Furthermore, blood-only immunophenotyping without paired tumor sampling and baseline differences between groups in some CD4+ subpopulations (higher baseline Th2 and Treg fractions in cryotherapy) should also be acknowledged as a potential confounder, despite randomization. However, the collection of paired tumor-blood samples at several time points would provide additional risks to the patients, and is currently not feasible in clinical practice. Finally, the discordance between tumor radiological response and overall survival requires further investigation and might be related to several factors, including the heterogeneous tumor mutational landscape and acquired tumor resistance.

## 4. Materials and Methods

### 4.1. Study Design, Setting, and Treatment Procedures

This prospective, randomized, controlled study, enrolling patients with metastatic NSCLC eligible for cryotherapy and first-line systemic immunotherapy with or without chemotherapy, was conducted at the Lithuanian University of Health Sciences Hospital, Kauno Klinikos. Participants were enrolled between 27 February 2023 and 27 August 2025.

The study was conducted in accordance with the Declaration of Helsinki, approved by the Kaunas Regional Biomedical Research Ethics Committee (Protocol BE-2-14, 18 January 2023) and registered at ClinicalTrials.gov [NCT06000358]. Written informed consent was obtained from all participants prior to enrolment.

The inclusion criteria, randomization, and study interventions were described earlier and will be covered briefly [40]. Adult patients who had histologically confirmed metastatic NSCLC, no activating epidermal growth factor receptor (EGFR) or anaplastic lymphoma kinase (ALK) gene mutations, and known PD-L1 expression on tumor cells were eligible if they scored 0 to 1 according to the ECOG performance status score, had at least one pulmonary lesion reachable via flexible bronchoscopy, and received no surgery, radiotherapy, or chemotherapy in the last 12 months, nor prior immunotherapy. Patients with brain metastases were permitted to enroll after Gamma Knife radiosurgery, provided they were stable enough for systemic treatment. Patients were excluded if they were unable or unwilling to undergo bronchoscopy; were previously diagnosed with autoimmune or immunosuppressive diseases, hepatitis B or C, active human immunodeficiency virus, or pulmonary tuberculosis infection; or were receiving immunosuppressive drugs or systemic corticosteroids (with prednisolone-equivalent doses exceeding 10 mg daily).

Participants were randomized in a 1:1 ratio to either the cryotherapy or control group. The cryotherapy group received bronchoscopic cryotherapy followed by standard-of-care first-line systemic therapy with pembrolizumab monotherapy for PD-L1 TPS ≥ 50%, or pembrolizumab plus platinum-based chemotherapy for PD-L1 TPS < 50%. The control group received only the standard-of-care first-line systemic therapy as described earlier. Patients who had an unsuccessful cryotherapy procedure, due to the inability to correctly position the cryoprobe within the lesion, were reassigned to the control group.

Bronchoscopic cryotherapy was performed via flexible bronchoscope (Olympus Corporation, Tokyo, Japan) 7 (±1) days prior to initiation of systemic therapy, with cryotherapy (Erbe Elektromedizin GmbH, Tübingen, Germany) performed under visual (for endobronchial cryotherapy) or radial endobronchial ultrasound (EBUS) and fluoroscopy (for transbronchial cryotherapy) guidance. Carbon dioxide (CO_2_) was used as the cryogenic gas for 60 s. Afterwards, passive cryoprobe thawing for 60 s followed. The cooling–thawing stages were repeated for a total of 3 times to maximize tumor cell destruction in the treated area.

Statistical power analysis was performed, which showed that a sample of 76 patients would have a power of 80% to detect an increase in ORR from 20% in the control group to 47% in the cryotherapy group with *p* < 0.05.

### 4.2. Radiological Assessment

Tumor imaging was performed using computed tomography (CT) every 3 cycles of systemic therapy. Response was assessed according to RECIST 1.1 criteria to determine cases of complete response (CR), partial response (PR), stable disease (SD), or progressive disease (PD). Cases where a clear response could not be interpreted by the radiologist were discussed in a multidisciplinary tumor board meeting.

### 4.3. Blood Collection, Processing, and Flow Cytometry

For the cryotherapy group, the baseline blood sample was collected right before moving the patient to the bronchoscopy suite for bronchoscopic cryotherapy. For the control group baseline, as well as for both groups at weeks 3 and 6, blood collections were performed immediately before immunotherapy administration. The sampling times were chosen to provide comparisons to studies that use local lung tumor destruction with immunotherapy, such as CRYOMUNE [NCT04339218].

Blood was collected in ethylenediaminetetraacetic acid (EDTA) tubes. Peripheral blood mononuclear cells (PBMCs) were isolated using Lymphoprep density-gradient centrifugation and analyzed from fresh samples using a BD FACSLyric flow cytometer. Antibodies included: CD3 (FITC), CD4 (PE-Cy7), CD8 (APC-Cy7), T-bet (PerCP-Cy5.5), GATA3 (PE), and CD25 (PE) (BD Pharmingen, Franklin Lakes, NJ, USA); FOXP3 (BV421) (BioLegend, San Diego, CA, USA); RORγt (APC) (Miltenyi Biotec, Bergisch Gladbach, Germany). Because T-bet, GATA3, RORγt, and FOXP3 are intracellular targets, cells were permeabilized using the Foxp3/Transcription Factor Staining Buffer Set (eBioscience, San Diego, CA, USA).

**Figure 10:**
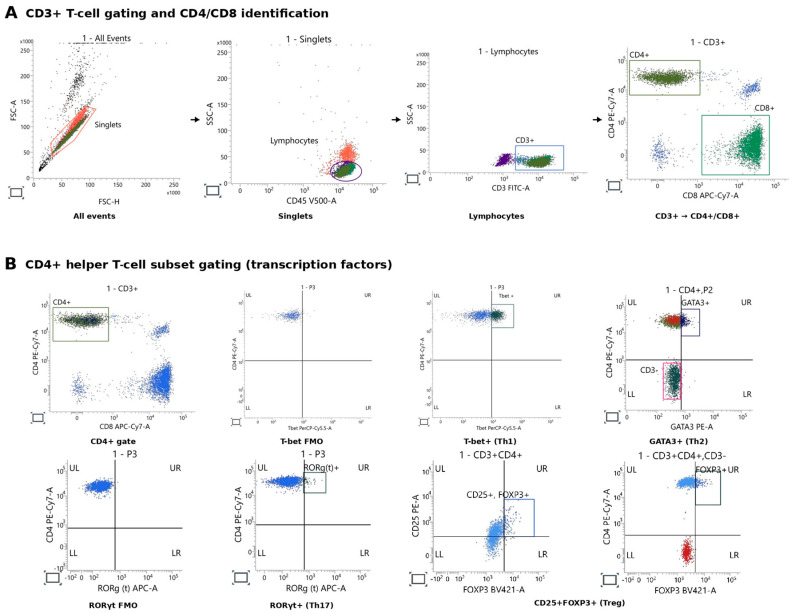
Flow-cytometry gating strategy for peripheral blood T-cell populations: (**A**) sequential gating of peripheral blood mononuclear cells to identify CD3+ T cells and CD4+/CD8+ subsets; (**B**) CD4+ T-cell subset gating—T-bet+ (Th1), GATA3+ (Th2), RORγt+ (Th17), and CD25+FOXP3+ (Treg).

The gating strategy for CD4+ T cells consisted of the following steps: exclusion of doublets → SSC/CD45 gate → CD3+ lymphocytes (T cells) → CD4+ T cells. Following this, CD4+ T cells expressing T-bet were identified using fluorescence minus one (FMO) control; CD4+ T cells expressing GATA3 were identified in comparison with non-T cells (CD3- lymphocytes); CD4+ T cells expressing RORγt were identified using FMO control; CD4+ T cells expressing CD25+ were identified using fluorescence minus one (FMO) control and FOXP3+ in comparison with non-T cells (CD3- lymphocytes). The gating strategy for CD8+ T cells consisted of the following steps: exclusion of doublets → SSC/CD45 gate → CD3+ lymphocytes (T cells) → CD8+ T cells (Figure 10). Data were analyzed using BD FACSuite software version 1.5.

### 4.4. Statistical Analysis

Continuous variables are presented as median (IQR) and categorical variables as n (%). For continuous variables, between-group comparisons at each time point were performed using the Mann–Whitney U test, while within-group changes between baseline, week 3, and week 6 were assessed using Wilcoxon’s signed-rank test. Categorical variables were evaluated using Fisher’s exact test or Pearson’s Chi-square tests. The Kaplan–Meier method was used to calculate median PFS and OS. Univariate and multivariable Cox regression analysis were used in calculating predictors of PFS and OS. Survival analysis was performed in the intention-to-treat population, while flow cytometry analysis was performed for patients who could provide blood samples at the specified time points. Patients who deteriorated too much to continue treatment or died were removed from the flow cytometry analysis. Exact two-tailed *p*-values were used, with *p*-values < 0.05 considered statistically significant. Statistical analyses were performed using SPSS version 30.0.0.0 and plotted using GraphPad Prism version 10.6.1.

CD4+ and CD8+ T cells are reported as a proportion of CD3+ T cells, meanwhile CD4+Tbet+ T cells (T helper 1, Th1), CD4+GATA3+ T cells (T helper 2, Th2), CD4+RORγt+ T cells (T helper 17, Th17), and CD4+CD25+FOXP3+ T cells (regulatory T cells, Tregs) are reported as a proportion of CD4+ T cells.

## 5. Conclusions

In patients with metastatic NSCLC receiving first-line pembrolizumab with or without chemotherapy, the addition of bronchoscopic cryotherapy was associated with a detectable peripheral immune remodeling as a decrease in circulating CD4+ T cells and an increase in CD8+ T cells by week 6, as well as a decline in circulating CD25+FOXP3+CD4+ regulatory T cells by week 3. These CD4+, CD8+, and Treg cell changes were also observed to be most prevalent in responders. Clinically, cryotherapy was associated with a higher objective response rate, while survival outcomes showed a numerical but non-significant improvement. Treg decline was also associated with improved PFS in regression analyses, and PD-L1 TPS was an independent factor for both PFS and OS. These findings support further evaluation of bronchoscopic cryotherapy as an immune-modulating addition to pembrolizumab-based therapy, and highlight Treg dynamics as a candidate biomarker for treatment benefit.

## Figures and Tables

**Table 1:** Patient characteristics.

Characteristic	Cryotherapy Group (n = 34)	Control Group (n = 42)	*p*-Value
Median age—years (IQR)	65.0 (61.0–70.0)	65 (60.0–73.0)	0.687
Gender—n (%)			
Male	26 (76.5)	33 (78.6)	1.000
Female	8 (23.5)	9 (21.4)	
Smokers—n (%)	30 (88.2)	38 (90.5)	1.000
ECOG performance status—n (%)			
0	9 (26.5)	11 (26.2)	1.000
1	25 (73.5)	31 (73.8)	
Histology—n (%)			0.248
Adenocarcinoma	16 (47.1)	26 (61.9)	
Squamous cell	18 (52.9)	16 (38.1)	
PD-L1 tumor proportion score—n (%)			
≥50%	15 (44.1)	16 (38.1)	0.801
1–49%	9 (26.5)	10 (23.8)	
<1%	10 (29.4)	16 (38.1)	
First-line systemic treatment—n (%)			
Pembrolizumab monotherapy	15 (44.1)	16 (38.1)	0.644
Pembrolizumab + chemotherapy	19 (55.9)	26 (61.9)	
Metastatic sites—n (%)			
Pleura	10 (29.4)	14 (33.3)	0.806
Bones	12 (35.3)	9 (21.4)	0.205
Contralateral lung	9 (26.5)	7 (16.7)	0.398
Liver	4 (11.8)	9 (21.4)	0.363
Adrenal glands	5 (14.7)	7 (16.7)	1.000
Brain	3 (8.8)	6 (14.3)	0.723
Kidneys	2 (5.9)	1 (2.4)	0.584
Pancreas	1 (2.9)	2 (4.8)	1.000
Skin	2 (5.9)	1 (2.4)	0.584

n—number of patients; IQR—interquartile range; ECOG—Eastern Cooperative Oncology Group; PD-L1—programmed death-ligand 1.

**Table 2:** Peripheral blood CD4+ and CD8+ T cells over time.

Population (% of CD3+)	Cryotherapy Group			Control Group		
	Baseline (n = 34)	Week 3 (n = 30)	Week 6 (n = 28)	Baseline (n = 42)	Week 3 (n = 35)	Week 6 (n = 35)
CD4+	60.00 (51.00–65.25)	57.00 (50.75–65.00)	52.00 * (43.00–57.75)	58.00 (40.75–70.25)	60.00 (43.00–70.00)	54.00 (41.00–71.00)
CD8+	33.00 (27.75–39.50)	35.50 (29.25–41.50)	40.00 * (35.25–45.50)	34.50 (27.75–50.00)	35.00 (26.00–49.00)	36.00 (23.00–51.00)

Values provided as median (IQR). * *p* < 0.05 between baseline and week 6.

**Table 3:** Peripheral blood Th1, Th2, Th17, and Tregs over time.

Population (% of CD4+)	Cryotherapy Group			Control Group		
	Baseline (n = 34)	Week 3 (n = 30)	Week 6 (n = 28)	Baseline (n = 42)	Week 3 (n = 35)	Week 6 (n = 35)
CD4+T-bet+ (Th1)	6.57 (3.18–13.51)	5.36 (1.91–16.95)	8.11 (2.43–16.28)	6.84 (3.15–13.18)	6.00 (1.72–11.73)	5.00 (1.60–9.64)
CD4+GATA3+ (Th2)	24.75 ^‡^ (17.56–33.91)	21.00 (14.30–29.15)	22.09 (14.98–29.91)	17.03 ^‡^ (8.41–27.36)	18.00 (10.00–28.82)	19.72 (12.21–24.34)
CD4+RORγt+ (Th17)	3.12 (1.93–4.81)	3.11 (2.21–4.52)	4.06 (1.17–6.25)	2.70 (1.18–5.09)	2.80 (1.10–4.60)	3.48 (2.00–5.30)
CD4+CD25+ FOXP3+ (Treg)	8.36 ^‡^ (5.17–13.78)	6.44 ^#^ (4.23–10.30)	5.77 (3.69–11.04)	5.20 ^‡^ (3.29–9.58)	6.69 (3.09–11.00)	5.27 (3.35–9.34)

Values provided as medians (IQR). ^#^ *p* < 0.05 between baseline and week 3, ^‡^ *p* < 0.05 between groups.

**Table 4:** Univariate and multivariable Cox analyses of progression-free survival and overall survival.

	Univariate PFS	Multivariable PFS		Univariate OS	Multivariable OS	
	*p*-Value	HR (95% CI)	*p*-Value	*p*-Value	HR (95% CI)	*p*-Value
Age	0.414			0.585		
<65 years		1 (reference)			1 (reference)	
≥65 years		1.17 (0.51–2.69)	0.719		1.52 (0.56–4.14)	0.414
Gender	0.586			0.522		
Male		2.31 (0.71–7.51)	0.164		2.21 (0.49–9.86)	0.300
Female		1 (reference)			1 (reference)	
Smoking status	0.792			0.950		
Never smoker		1 (reference)			1 (reference)	
Former/Current		0.44 (0.12–1.69)	0.232		0.26 (0.53–1.24)	0.090
ECOG	0.973			0.513		
0		1 (reference)			1 (reference)	
1		1.05 (0.39–2.81)	0.928		1.51 (0.50–4.55)	0.463
Histology	0.859			0.376		
Adenocarcinoma		0.90 (0.37–2.20)	0.818		1.09 (0.35–3.41)	0.882
Squamous cell		1 (reference)			1 (reference)	
PD-L1 TPS	0.334			0.217		
≥50%		0.34 (0.14–0.82)	0.016		0.26 (0.08–0.79)	0.018
1–49%		0.85 (0.36–2.03)	0.721		0.58 (0.20–1.66)	0.310
<1%		1 (reference)			1 (reference)	
Local treatment	0.243			0.349		
Cryotherapy		0.52 (0.19–1.36)	0.181		0.71 (0.23–2.19)	0.551
Control		1 (reference)			1 (reference)	
T-cell changes at week 3						
CD4+ increase	0.530	1 (reference)		0.968	1 (reference)	
CD4+ decrease		0.57 (0.19–1.68)	0.307		0.45 (0.12–1.76)	0.252
CD8+ increase	0.795	1 (reference)		0.140	1 (reference)	
CD8+ decrease		0.75 (0.30–1.89)	0.546		0.31 (0.15–3.05)	0.123
Treg increase	0.037	1 (reference)		0.272	1 (reference)	
Treg decrease		0.26 (0.09–0.75)	0.012		0.49 (0.15–1.64)	0.246
T-cell changes at week 6						
CD4+ increase	0.941	1 (reference)		0.731	1 (reference)	
CD4+ decrease		1.03 (0.21–5.19)	0.969		0.99 (0.16–6.36)	0.998
CD8+ increase	0.320	1 (reference)		0.423	1 (reference)	
CD8+ decrease		0.33 (0.09–1.26)	0.1406		0.67 (0.15–3.05)	0.605
Treg increase	0.970	1 (reference)		0.988	1 (reference)	
Treg decrease		3.10 (0.91–10.53)	0.069		2.01 (0.44–9.25)	0.366

PFS—progression-free survival; OS—overall survival; 95% CI—95% confidence interval; ECOG—Eastern Cooperative Oncology Group; TPS—tumor proportion score.

**Table 5:** Best overall response rate by study group.

	Cryotherapy Group (n = 34)	Control Group (n = 42)	Total (n = 76)
Partial response—n (%)	14 (41.2)	7 (16.7)	21 (27.6)
Stable disease—n (%)	11 (32.4)	18 (40.5)	29 (38.2)
Progressive disease—n (%)	9 (26.5)	17 (40.5)	26 (34.2)

n—number of patients.

**Table 6:** Peripheral blood T cells over time according to tumor radiological response.

	Baseline	Week 3	Week 6
Partial response	(n = 21)	(n = 21)	(n = 21)
CD4+ (% of CD3+)	57.00 (49.00–64.50)	55.50 (47.00–63.00)	52.00 * (43.75–62.25)
CD8+ (% of CD3+)	36.00 (32.00–43.00)	40.00 (30.50–45.25)	42.00 (30.75–46.00)
CD4+CD25+FOXP3+ (Treg) (% of CD4+)	8.99 (6.38–13.90)	6.38 ^#^ (3.42–11.83)	7.14 (3.86–9.40)
Stable disease	(n = 29)	(n = 29)	(n = 29)
CD4+ (% of CD3+)	58.00 (44.00–65.75)	57.50 (45.50–65.25)	54.00 (41.00–65.00)
CD8+ (% of CD3+)	34.50 (27.25–49.50)	35.00 (30.50–45.00)	40.00 (27.00–51.00)
CD4+CD25+FOXP3+ (Treg) (% of CD4+)	6.46 (4.02–9.13)	7.22 (3.30–10.84)	5.20 (3.68–12.00)
Progressive disease	(n = 26)	(n = 15)	(n = 13)
CD4+ (% of CD3+)	60.50 (54.50–68.00)	62.50 (47.00–68.50)	60.50 (42.00–69.00)
CD8+ (% of CD3+)	32.00 (25.50–37.25)	33.50 (26.00–45.00)	33.00 (26.00–49.50)
CD4+CD25+FOXP3+ (Treg) (% of CD4+)	5.13 (3.62–10.25)	6.40 (3.91–7.39)	5.12 (2.69–7.20)

Values provided as median (IQR). n—number of patients. ^#^ *p* < 0.05 between baseline and week 3, * *p* < 0.05 between baseline and week 6.

## Data Availability

The original contributions presented in this study are included in the article/Supplementary Materials. Further inquiries can be directed to the corresponding authors.

## Supplementary Materials

The following supporting information can be downloaded at: https://www.mdpi.com/article/10.3390/ijms27072927/s1.

## Abbreviations

The following abbreviations are used in this manuscript:

ALK	Anaplastic lymphoma kinase
CO2	Carbon dioxide
CT	Computed tomography
DCR	Disease control rate
EBUS	Endobronchial ultrasound
ECOG	Eastern Cooperative Oncology Group
EDTA	Ethylenediaminetetraacetic acid
EGFR	Epidermal growth factor receptor
FOXP3	Forkhead box protein P3
ICI	Immune checkpoint inhibitor
IFN-α	Interferon-α
IQR	Interquartile range
NSCLC	Non-small-cell lung cancer
ORR	Overall response rate
OS	Overall survival
PBMC	Peripheral blood mononuclear cell
PD	Progressive disease
PD-1	Programmed cell death protein 1
PD-L1	Programmed death-ligand 1
PFS	Progression-free survival
PS	Performance status
PR	Partial response
RECIST 1.1	Response Evaluation Criteria In Solid Tumors version 1.1
SD	Stable disease
STING	Stimulator of interferon genes
TGF-β	Transforming growth factor-β
TPS	Tumor proportion score
Treg	Regulatory T cell
