# Supplementary material for: Longitudinal Analysis of Peripheral Blood CD4+ T-Cell Profiles and Clinical Outcomes in Metastatic Non-Small-Cell Lung Cancer Patients Following Bronchoscopic Cryotherapy and Pembrolizumab-Based Therapy
Source: Int J Mol Sci. 2026 Mar 24;27(7):2927. doi: 10.3390/ijms27072927 (PMC13073585; doi:10.3390/ijms27072927)
Supplement: Supplementary file 1 [file ijms-27-02927-s001.zip › ijms-4196988-supplementary.pdf]

Supplemental Table S1. Baseline peripheral blood T cells in different clinical groups

	<65 years (n=37)	≥65 years (n=39)	
CD4+ (% of CD3+)	60.00 (52.50–68.50)	58.00 (44.25–66.00)	
CD8+ (% of CD3+)	33.00 (28.00–38.50)	34.00 (27.00–46.50)	
CD4+T-bet+ (Th1) (% of CD4+)	6.70 (2.45–12.47)	6.70 (3.70–14.28)	
CD4+GATA3+ (Th2) (% of CD4+)	23.99 (12.21–35.00)	19.00 (13.00–28.41)	
CD4+RORγt+ (Th17) (% of CD4+)	3.11 (1.43–5.41)	2.90 (1.20–4.44)	
CD4+CD25+FOXP3+ (Treg) (% of CD4+)	5.98 (3.13–11.81)	7.87 (4.76–9.21)	
	Male (n=59)	Female (n=17)	
CD4+ (% of CD3+)	60.00 (50.00–67.00)	57.00 (41.50–64.75)	
CD8+ (% of CD3+)	33.00 (27.75–43.50)	36.00 (28.50–50.00)	
CD4+T-bet+ (Th1) (% of CD4+)	6.35 (2.91–13.33)	9.20 (5.28–13.58)	
CD4+GATA3+ (Th2) (% of CD4+)	20.49 (14.00–33.55)	17.70 (11.39–27.27)	
CD4+RORγt+ (Th17) (% of CD4+)	2.94 (1.29–5.15)	3.00 (2.02–4.44)	
CD4+CD25+FOXP3+ (Treg) (% of CD4+)	7.56 (3.74–11.90)	4.91 (3.82–8.60)	
	Former / current smoker (n=68)	Never smoker (n=8)	
CD4+ (% of CD3+)	60.00 (47.50–66.00)	58.00 (41.00–71.50)	
CD8+ (% of CD3+)	33.50 (28.00–45.00)	34.00 (24.25–47.00)	
CD4+T-bet+ (Th1) (% of CD4+)	6.70 (3.06–13.25)	7.82 (4.62–14.44)	
CD4+GATA3+ (Th2) (% of CD4+)	20.45 (15.11–32.00)	11.39 (7.72–20.62)	
CD4+RORγt+ (Th17) (% of CD4+)	3.12 (1.43–4.96)	2.09 (1.02–5.12)	
CD4+CD25+FOXP3+ (Treg) (% of CD4+)	7.25 (3.85–11.34)	5.86 (2.15–8.85)	
	ECOG PS 0 (n=20)	ECOG PS 1 (n=56)	
CD4+ (% of CD3+)	62.75 (53.75–69.50)	58.00 (45.50–66.00)	
CD8+ (% of CD3+)	33.00 (25.75–42.00)	34.50 (28.00–46.00)	
CD4+T-bet+ (Th1) (% of CD4+)	5.79 (2.90–8.83)	7.50 (3.42–14.22)	
CD4+GATA3+ (Th2) (% of CD4+)	19.40 (11.75–36.48)	20.00 (13.11–29.70)	
CD4+RORγt+ (Th17) (% of CD4+)	3.68 (1.32–6.74)	2.83 (1.43–4.41)	
CD4+CD25+FOXP3+ (Treg) (% of CD4+)	5.89 (4.10–9.21)	7.71 (3.76–11.95)	
	Adenocarcinoma (n=42)	Squamous cell (n=34)	
CD4+ (% of CD3+)	51.00 (46.50–66.00)	60.00 (49.00–67.75)	
CD8+ (% of CD3+)	34.50 (29.00–46.50)	33.00 (26.50–43.50)	
CD4+T-bet+ (Th1) (% of CD4+)	6.99 (2.78–13.10)	6.43 (3.49–14.52)	
CD4+GATA3+ (Th2) (% of CD4+)	21.51 (11.89–30.07)	19.67 (14.75–33.64)	
CD4+RORγt+ (Th17) (% of CD4+)	3.65 (1.53–5.64)	2.09 (1.20–3.98)	
CD4+CD25+FOXP3+ (Treg) (% of CD4+)	6.95 (4.47–11.11)	7.34 (3.62–11.25)	
	Pembrolizumab monotherapy (n=31)	Pembrolizumab +chemotherapy (n=45)	
CD4+ (% of CD3+)	58.00 (45.00–65.50)	60.00 (49.50–68.25)	
CD8+ (% of CD3+)	36.00 (27.00–48.00)	33.00 (28.00–44.75)	
CD4+T-bet+ (Th1) (% of CD4+)	5.17 (2.00–10.12)	8.00 (3.63–13.80)	
CD4+GATA3+ (Th2) (% of CD4+)	20.83 (15.00–33.19)	19.33 (12.19–32.00)	
CD4+RORγt+ (Th17) (% of CD4+)	2.90 (1.70–4.59)	3.11 (1.33–5.36)	
CD4+CD25+FOXP3+ (Treg) (% of CD4+)	6.80 (4.76–8.70)	8.53 (3.74–11.81)	
	PD-L1 TPS <1% (n=26)	PD-L1 TPS 1-49% (n=19)	PD-L1 TPS ≥50% (n=31)
CD4+ (% of CD3+)	60.00(46.00–69.00)	60.00 (51.00–66.00)	58.00 (45.00–65.50)
CD8+ (% of CD3+)	33.00 (27.00–46.00)	34.00 (29.25–44.00)	36.00 (27.00–48.00)
CD4+T-bet+ (Th1) (% of CD4+)	6.43 (3.19–13.10)	9.41 (6.23–14.28)	5.17 (2.00–10.12)
CD4+GATA3+ (Th2) (% of CD4+)	19.67 (8.41–32.39)	18.00 (13.42–32.00)	20.83 (15.00–33.19)
CD4+RORγt+ (Th17) (% of CD4+)	2.16 (1.18–4.48)	4.23 (1.55–6.80)	2.90 (1.70–4.59)
CD4+CD25+FOXP3+ (Treg) (% of CD4+)	7.49 (3.10–11.77)	8.53 (3.80–11.96)	6.80 (4.76–8.70)

Values provided as medians (IQR). ECOG PS—Eastern Cooperative Oncology Group performance status; TPS—tumor proportion score.

Supplemental Table S2. Univariate and multivariable Cox analyses of progression-free survival and overall survival (continued from Table 4).

	Univariate PFS	Multivariable PFS		Univariate OS	Multivariable OS	
	p-value	HR (95% CI)	p-value	p-value	HR (95% CI)	p-value
T cell changes at week 3						
Th1 increase	0.904	1 (reference)		0.314	1 (reference)	
Th1 decrease		0.64 (0.21–1.93)	0.428		1.65 (0.48–5.64)	0.427
Th2 increase	0.962	1 (reference)		0.335	1 (reference)	
Th2 decrease		0.94 (0.37–2.37)	0.899		0.41 (0.13–1.26)	0.120
Th17 increase	0.728	1 (reference)		0.883	1 (reference)	
Th17 decrease		1.35 (0.59–3.13)	0.479		1.54 (0.54–4.43)	0.422
T cell changes at week 6						
Th1 increase	0.985	1 (reference)		0.677	1 (reference)	
Th1 decrease		2.98 (0.83–10.73)	0.094		1.49 (0.40–5.55)	0.546
Th2 increase	0.398	1 (reference)		0.143	1 (reference)	
Th2 decrease		1.10 (0.33–3.65)	0.883		0.80 (0.19–3.44)	0.769
Th17 increase	0.522	1 (reference)		0.745	1 (reference)	
Th17 decrease		1.50 (0.65–3.46)	0.338		1.75 (0.67–4.58)	0.257

PFS—Progression-free survival; OS—overall survival; 95% CI—95% confidence interval.

Supplemental Table S3. Peripheral blood T cells over time according to tumor radiological response

	Baseline	Week 3	Week 6
Partial response	(n = 21)	(n = 21)	(n = 21)
CD4+ (% of CD3+)	57.18 (49.34–64.66)	55.50 (47.13–62.90)	52.00 * (43.76–62.25)
CD8+ (% of CD3+)	35.94 (32.00–43.07)	40.00 (30.75–45.25)	42.00 (30.75–46.04)
CD4+T-bet+ (Th1) (% of CD4+)	7.35 (3.39–14.02)	5.57 (1.91–14.10)	8.82 (2.90–14.48)
CD4+GATA3+ (Th2) (% of CD4+)	23.95 (16.98–32.00)	24.25 (16.97–31.87)	21.98 (12.17–29.29)
CD4+RORγt+ (Th17) (% of CD4+)	3.21 (1.93–6.33)	3.46 (2.01–5.07)	4.06 (2.09–5.53)
CD4+CD25+FOXP3+ (Treg) (% of CD4+)	8.99 (6.38–13.90)	6.38 # (3.42–11.83)	7.14 (3.86–9.40)
Stable disease	(n = 29)	(n = 29)	(n = 29)
CD4+ (% of CD3+)	58.00 (44.00–65.73)	57.41 (45.55–65.22)	54.00 (41.00–65.00)
CD8+ (% of CD3+)	34.50 (27.25–49.50)	35.28 (30.50–45.00)	40.00 (27.00–51.00)
CD4+T-bet+ (Th1) (% of CD4+)	8.60 (5.58–13.87)	6.50 (4.91–13.79)	6.30 (3.70–15.20)
CD4+GATA3+ (Th2) (% of CD4+)	19.66 (11.70–28.03)	18.56 (9.67–28.41)	19.72 (12.69–22.85)
CD4+RORγt+ (Th17) (% of CD4+)	3.05 (1.17–4.50)	2.83 (1.46–4.00)	4.06 (2.97–5.40)
CD4+CD25+FOXP3+ (Treg) (% of CD4+)	6.46 (4.02–9.13)	7.22 (3.30–10.84)	5.20 (3.68–12.00)
Progressive disease	(n = 26)	(n = 15)	(n = 13)
CD4+ (% of CD3+)	60.50 (54.61–68.03)	62.47 (47.00–68.41)	60.56 (42.06–69.00)
CD8+ (% of CD3+)	32.16 (25.69–37.23)	33.67 (26.36–45.00)	33.12 (26.22–49.44)
CD4+T-bet+ (Th1) (% of CD4+)	4.25 (1.60–9.75)	1.80 (0.65–7.26)	3.59 (1.07–10.91)
CD4+GATA3+ (Th2) (% of CD4+)	19.40 (11.25–36.97)	16.27 (13.43–21.20)	19.56 (13.30–26.09)
CD4+RORγt+ (Th17) (% of CD4+)	2.75 (1.40–4.69)	2.73 (2.19–3.28)	2.36 (1.89–4.10)
CD4+CD25+FOXP3+ (Treg) (% of CD4+)	5.13 (3.62–10.25)	6.40 (3.91–7.39)	5.12 (2.69–7.20)

Values provided as medians (IQR). # $p<0.05$ between baseline and week 3, * $p<0.05$ between baseline and week 6.
